# From the chemical imbalance to the power imbalance: A psychiatry trainee’s perspectives on service-user supervision

**DOI:** 10.1177/10398562231191695

**Published:** 2023-08-04

**Authors:** Chiranth Bhagavan, Sarah Gordon, Frederick Sundram

**Affiliations:** Department of Psychological Medicine, School of Medicine, 62710 University of Auckland, Auckland, New Zealand; Department of Psychological Medicine, School of Medicine and Health Sciences, 2495 University of Otago, Wellington, New Zealand; Department of Psychological Medicine, School of Medicine, 62710 University of Auckland, Auckland, New Zealand

**Keywords:** service-user supervision, recovery, human rights, supported decision-making, psychiatric education

## Abstract

**Objective:**

This paper describes perspectives and insights of a trainee’s experience of service-user supervision. This includes the background to this novel approach, outlining its process and content, key themes arising, applications in practice, limitations of the approach, and future considerations.

**Conclusions:**

Service-user supervision promotes education and experiences at this important stage of professional development and can promote clinical, cultural, and systemic changes required to support the paradigm shift towards recovery-oriented and human rights-based practice.

Article 12 of the United Nations Convention on the Rights of Persons with Disabilities (CRPD), which New Zealand and Australia ratified in 2008, affirms the equal recognition before the law and legal capacity of persons with disabilities. The CRPD Committee interpreted this as a shift from ‘substitute decision-making’ to ‘supported decision-making’.^1^ In New Zealand, the Ministry of Health, via He Ara Oranga (the Mental Health Inquiry) in 2019, committed towards practice that is aligned with recovery-oriented and human rights-based approaches such as supported decision-making and the repeal and replacement of the Mental Health Act.^2^

In 2011, the University of Otago, Wellington, Department of Psychological Medicine committed to a reform: a service-user-led educational programme for medical students and psychiatry trainees to promote recovery and human rights. A thematic analysis of data collected by psychiatry trainees following this educational program showed this prompted a paradigm shift, trainees had more promise to reduce restrictive practices, but felt limited by power differentials and systemic challenges.^3^ From 2018, the programme offered optional service-user supervision to psychiatry trainees.

In reviewing the literature, we identified only one article describing service-user supervision in another service to healthcare professionals – group-based supervision for nursing students.^4^ This described initial reservations from students in accepting service-users as supervisors to potential benefits in improved awareness and confidence in promoting person-centred principles. However, the literature review identified no other services that offered this for psychiatry trainees.

The supervisor and first trainee from this University of Otago programme to pilot service-user supervision noted benefits in facilitating recovery-oriented approaches and challenges in implementing these in practice due to power dynamics and hierarchical medical structures.^5^

This article describes perspectives and insights of service-user supervision of another psychiatry trainee (CB) from this programme, with reflections provided by his service-user supervisor (SG) and academic supervisor (FS). This piece expands on the structure and process of service-user supervision, key lessons learned, applications in practice, limitations, and future considerations. It is argued that providing service-user supervision to psychiatry trainees promotes education and experiences during this important stage of professional development and supports changes required for the paradigm shift towards recovery-oriented and human rights-based practice.

## My experience (written from the perspective of CB)

By my second year as a psychiatry trainee in Wellington in 2019, I had been exposed to numerous ethical considerations and socio-political tensions inherent in our clinical practice, reinforced by the CRPD and He Ara Oranga. I then sought service-user supervision to process these internal conflicts.

The supervisor and I met monthly, one-to-one, for 1 hour. Protected time for supervision was supported by the local training programme and clinical service. The time and day were kept consistent to maintain the ‘frame’, book a private room, and plan service and training needs outside this.

Regarding content, I would bring cases which left me with ethical tensions spanning themes of potentially coercive treatment, difficulties with therapeutic alliance, or challenging service dynamics when implementing recovery-oriented practice. In turn, the supervisor would reflect on similar cases based on their lived experience, collective knowledge from peers, and experience from consumer advocacy work across individual and systemic levels to convey service-user-focused perspectives regarding the situation and interests of clients discussed. Building on this, recovery principles and human rights relevant to the case were identified and explored, informing potential changes in practice.

The supervisory relationship developed over time towards a position of mutual respect, enabling richer reflection and growth. The interactions initially challenged my pre-existing clinical and ethical positions, evoking difficult emotions and resistance towards these divergent perspectives. However, reflection on these evoked emotions prompted awareness of my own stigma and unconscious biases. I was then more open to these challenges, facilitating a safe space for this exchange of ideas and learning.

Key lessons and applications towards recovery-oriented and human rights-based practice arising from this supervision are outlined in Table 1.

**Table 1.** Key Themes Arising From Service-User Supervision

Theme	Discussion in supervision	Actions and applications	Changes in recovery-oriented practice arising from these actions
Supported decision-making	• Viewing matters from a service user perspective	• Modifying history taking from standardised templates towards richer explorations of the client’s values and narratives	• Enabled deeper and broader treatment discussions
	• Moving from the ‘best interests’ paradigm (commonly associated with substitute decision-making) to the ‘wills and preferences’ paradigm (associated with supported decision-making)	• Advanced care planning	• Empowered the client’s input and autonomy
		• Involving family more	
		• Providing more treatment options	
Power imbalances	• Acknowledging our sense of powerlessness as trainees working under a system with competing pressures and expectations^3^	• First acknowledging this imbalance to raise conscious awareness and identify strategies to lessen this imbalance	• Helped relax any tensions
	• Reflecting that if we felt this powerlessness, imagine how the client felt?	• Whakawhanaungatanga (building relationships and connectedness) with clients	• Supported clients to feel comfortable and share their story with me
	• Exemplified when discussing cases involving coercive treatments such as use of the Mental Health Act	• Therapeutic self-disclosure about aspects of my upbringing, family, and hobbies	• Greater trust and therapeutic alliance
			• Enabled clients to better understand and participate
Epistemic injustice	• Understanding that this is when a client’s engagement in epistemic practices – such as providing knowledge or interpreting their experience – is undermined^6^	• Reflection in supervision for exposing and exploring these unconscious biases	• Clients felt better heard and understood
	• Can arise via a clinician’s overemphasis on one model at the expense of others – such as biological or psychodynamic – and how stigma and unconscious biases can dismiss a client’s perspective and needs	• Implementing ‘shared’ clinic letters and formulations. This involved sharing my written assessment with the client and revising as necessary in light of new information and the client’s perspective, prior to finalising what was then a documented shared understanding and providing the client a copy	• Enabled greater input from clients
	• Harms may include undertreatment, diagnostic overshadowing, distrust, and disempowerment		• Led to greater hope and confidence in their recovery
	• Case examples included where my disagreement in clients’ self-framing of their distress or level of risk rendered potential iatrogenic harm by undermining their knowledge, invalidating their experience, reinforcing inadequacy and inferiority, and exacerbating their difficulties		
Trauma-informed care	• The role of trauma in underpinning psychopathology and functioning, particularly via stigma, discrimination, and previous negative experiences with psychiatric services	• Providing options for conducting their assessment such as home-based reviews and treatment	The guiding principles of trauma-informed care^7^ were adhered to, including:
	• The potential for re-traumatisation was illustrated when discussing case examples involving the use of seclusion, restraint, and overuse of medication	• Involving family, peer support, and cultural-liaison services	• Ensuring the client’s sense of psychological and physical safety
		• Providing a range of management options from biological, psychological, social, and cultural approaches	• Improving trust and rapport
		• Listening to and learning from their previous negative experiences, thereby facilitating different approaches	• Promoting the client’s voice, input, choice, and control
		• Exploring alternatives to seclusion and restraint with staff members	• Acknowledging and addressing issues arising from stigma, historical trauma, and discrimination

The most recurring theme brought to supervision was regarding clients who had previous negative experiences with psychiatric services, often pertaining to predominantly biomedical and involuntary treatment approaches.

Supervision exposed the prominence of trauma in these clients via medication overuse, seclusion, and restraint and how this intersected with pervasive trauma arising from individual events, stigma, and historical trauma, particularly in disadvantaged groups such as Māori. The role of trauma in underpinning their psychopathology and potential for re-traumatisation via restrictive clinical practices become apparent.^7^ Supervision deepened my understanding of a client’s sense of powerlessness when exploring these previous restrictive practices, drawing parallels to the powerlessness we feel as clinicians due to systemic challenges, such as the difficulty terminating seclusion due to staffing shortages. Supervision exposed unconscious biases in cases where I disagreed with a client’s self-framing of their difficulties, which, when coupled with these power imbalances, compound the potential harm for further disempowerment and distrust via epistemic injustice.^6^ For example, my disagreement with one client’s self-framing of dissociative identity disorder, possibly influenced by public scepticism of the diagnosis, served as unhelpful by undermining their knowledge, trust, and hope in their recovery.

Greater emphasis was subsequently placed on establishing trust and rapport foremost, listening to their story, and exploring working with them differently. Trauma-informed and shared formulations, as described in Table 1, lessened these power imbalances and epistemic injustices, facilitating more collaborative insights and management approaches and promoting their input, trust, hope, and confidence in their recovery. Additional suggestions to allow trauma-informed, holistic, and supported decision-making from the outset included offering clients home-based assessments, inviting support persons, and bringing non-medical staff to facilitate a multi-disciplinary approach, promote their sense of safety, and mitigate fears of exclusively biomedical paradigms. A foundation of greater therapeutic alliance was more readily established, allowing exploration and agreement for a wider range of management options across the biopsychosocial spectrum, limiting the need for restrictive measures and promoting their choice and autonomy.

Supervision encouraged learning from clients’ previous negative experiences, thereby facilitating alternative, recovery-oriented, and human rights-based approaches which not only improved care and engagement during these encounters, but it also reinstated hope and optimism in clients towards psychiatric services for the future.

## Challenges and limitations (written from the perspective of CB)

Recovery-oriented and human rights-based suggestions frequently clashed with conventional attitudes. For example, in consultation-liaison rotations, I advocated for supported decision-making when providing capacity assessments, acknowledging its nuanced nature and providing time, involving family, and respecting relevant individual preferences. However, medical colleagues preferred immediate and binary yes or no capacity judgements to justify whether they could act based on their perspective of the client’s best interests, rather than supporting the individual’s will and preferences, leading to tensions between clinical teams.

Similarly, reflections from service-user supervision potentially contradicted biomedical evidence or senior clinical advice. For example, using the Mental Health Act or specific antipsychotics based on this advice or evidence against the client’s preferences was explored in supervision for clashing with supported decision-making principles.

These challenges prompted wider conversations regarding limitations of biomedical evidence at an individual level – recognising the need for integrated, epistemological approaches which balance the natural and social sciences inherent within psychiatric practice.^8^ Discussing these clashes within the safe and respectful space which supervision afforded enabled informed and considered decisions, incorporating this knowledge from different sources. In turn, the respectful dialogue modelled in supervision was applied with medical colleagues in consultation-liaison rotations – respecting their perspectives and preserving collegiality whilst advocating for clients’ rights. And whilst biomedical and senior clinical advice may support certain medications and the Mental Health Act at times, this need not strip clients of their autonomy entirely, for example, by providing different antipsychotic options and access to their unique supports, such as their own music or family visits, despite being involuntary inpatients. Supervision thereby explored strategies where even when the Mental Health Act is used, this process can enable some degree of supported decision-making by effectively communicating clients’ human rights, promoting choices and autonomy, and respecting dignity despite extant restrictions.

Further systemic challenges included existing risk-averse practices resistant to change. For example, inpatient psychiatric units resisted voluntary admissions despite my advocacy. In the wider context, I felt more vulnerable to public scrutiny and claims of negligence if adverse outcomes occurred because of person-centred approaches, such as voluntary-based treatments. Whereas I felt less scrutiny if involuntary measures were taken to mitigate risks.

Moreover, it is yet to be determined if shifts in power imbalances pose risks themselves. These may include reduced emphases on accountability and clinical governance, clinician dissatisfaction arising from perceived role-reversals, and possible adverse outcomes arising from less restrictive practices.

Resource constraints presented multiple limitations. These ranged from staffing shortages in inpatient units increasing pressure towards using seclusion, time pressures in emergency departments limiting opportunities for deeper explorations and shared clinic letters, and few psychosocial resources – limiting the provision of options and indirectly driving restrictive and biomedical approaches.

Whilst direct, clinical barriers could be explored and navigated as discussed, these more systemic limitations rendered moments of paralysis. However, they also served as additional drivers for systemic advocacy during positions of greater leadership and management in the future.

## Discussion

This article builds on the limited literature for service-user supervision by outlining its process and content, expanding on the recovery and human rights-based principles applied, and broadening the discussion about its limitations. There are several parallels to the existing literature, including initial reservations towards working with service users, benefits including improved awareness and motivation towards recovery-oriented and human rights-based practices, and systemic limitations when applying these changes.

Further, this article introduces the added benefit supervision confers beyond existing educational programs through facilitating deeper reflective practice, practical guidance for translational shifts in recovery-oriented and human rights-based practice, and support in navigating the numerous challenges.

Whilst the limitations need not preclude this approach, these remain important considerations to ensure balanced shifts in practice occur while maintaining recovery and human rights as central components of clinical care.

Psychiatric training involves exposure to complex clinical cases and active development and practice of clinical knowledge and skills. Therefore, insights from this supervision at this important stage of professional development convey the added potential for supporting changes in practice for the next generation of psychiatrists who have an important role in facilitating the paradigm shift towards recovery-oriented and human rights-based approaches.

Table 2 suggests important factors regarding trainee components, supervisor characteristics, and the supervisory relationship as identified by factor analysis.^9^ Attention to these factors would promote a safe, respectful, non-judgemental, yet challenging space, enabling these reflections and growth.

**Table 2.** Important Trainee, Supervisor, and Supervisory Relationship Components to Consider for Service-User supervision

Trainee components	Supervisor components	Supervisory relationship components^9^
• Stage of training	• Sufficient advocacy experience	• Safe base
° Offered across all stages	• Confidence	• Structure
° Greatest utility may be from stage 2 to allow for time to prompt this type of reflection • Commitment to engagement and application • Protected time	• Consistency • Supervision training	• Commitment • Reflective education • Role model • Formative feedback

Further considerations include defining the structure and process of supervision, timing, frequency, comparing individual versus group approaches, audio-visual access, supervisor recruitment, and practical implications regarding service impacts and its fit within the broader training program. Future studies measuring outcomes of this supervision including trainee satisfaction, supervisor satisfaction, client and whānau outcomes, and cost-benefit analyses will help inform the usefulness of this approach.

## Conclusion

As a result of the exposure to the benefits of service-user supervision, I demonstrate respect towards contributions from service-user advocates, promoting an environment of greater acceptance of recovery-oriented care and human-rights based approaches. I have also developed service-level advocacy, presenting on this topic to colleagues, managers, and leaders involved in implementing service changes. Service-user supervision has since been offered to and taken-up by consultant psychiatrists in Wellington, reflecting a positive reception from my advocacy.

Service-user supervision for psychiatric trainees can promote clinical, cultural, and systemic changes required to support the paradigm shift towards recovery-orientated and human rights-based practice.
